# Stakeholders’ perceptions of the synergy between intellectual property and open science: A cross-sectional survey

**DOI:** 10.12688/openreseurope.20782.1

**Published:** 2025-08-07

**Authors:** Gautam Sharma, Claire Fritz, Alessandra Baccigotti, Pavel Stoev, Marie Alavi, Lea Škorić, Frantzeska Papadopoulou Skarp, Julia Priess-Buchheit, Gustav Nilsonne

**Affiliations:** 1 Karolinska Institutet, Stockholm, Sweden; 2 Centre for Innovation Research (CIRCLE), Lund University, Lund, Sweden; 3 Eurice GmbH, Sankt Ingbert, Germany; 4 Alma Mater Studiorum – University of Bologna, Bologna, Italy; 5 Pensoft Publishers, Sofia, Bulgaria; 6 National Museum of Natural History at the Bulgarian Academy of Sciences, Sofia, Bulgaria; 7 Christian-Albrechts-Universität zu Kiel, Kiel, Germany; 8 University of Zagreb School of Medicine, Zagreb, Croatia; 9 Stockholm University, Stockholm, Sweden; 10 Swedish National Data Service, Gothenburg University, Gothenburg, Sweden

**Keywords:** intellectual property, open science, patents, knowledge valorisation, science policy

## Abstract

**Background:**

The potential for intellectual property (IP) and open science (OS) to be used together for knowledge valorisation is increasingly discussed within research policy, yet their practical alignment remains limited. While researchers and research managers often regard IP and OS as potentially complementary, uncertainty persists about how to integrate them effectively in real-world contexts.

**Methods:**

To explore current perceptions and practices, a cross-sectional online survey was conducted, yielding 177 valid responses from individuals involved in research and research management across a range of research-performing organisations, primarily within Europe.

**Results:**

Although respondents were generally optimistic about the possibility of combining IP and OS, concrete examples of successful synergy remain rare. A lack of clear institutional guidance, limited awareness of open licensing mechanisms, and uncertainty about concurrent implementation were identified as key barriers. In collaborative projects, especially those involving multiple partners, differing objectives further complicate efforts to coordinate IP and OS strategies. National differences in legal frameworks across EU member states were also seen as an obstacle to synergy. The roles of knowledge transfer offices and OS ambassadors were highlighted as particularly important for supporting researchers in navigating complex decisions about licensing and compliance. However, sustained engagement with these actors remains inconsistent.

**Conclusions:**

To enable greater complementarity between IP and OS, early agreement on approaches within project management is essential. Researchers would benefit from targeted training to enhance understanding of open licensing tools and practical considerations. In addition, policy measures such as recognising OS contributions in academic career evaluation and introducing grace periods for patent filing could provide valuable support. These steps may assist institutions in managing the balance between openness and protection more effectively, encouraging broader and more confident application of both frameworks in practice. The study’s findings should be interpreted with caution due to the small, purposive sample and potential response bias, which may limit generalisability.

## Plain language summary

This article looks at how researchers and other people involved in science think about two important concepts: intellectual property (IP) and open science (OS). IP includes things like patents and copyrights, which protect inventions and ideas. Open science is about sharing research freely so that more people can benefit from it. The study asked 177 people in the research community about their views and experiences with these two approaches. Most people believe that IP and OS can work well together, but in reality, they are not often used side by side. There are several reasons for this, including a lack of clear rules, not enough knowledge about how to combine them, and different goals among research partners. The study suggests that researchers should make early agreements about how to handle IP and OS when working together. It also recommends filing patents early to avoid problems with sharing research openly. To help researchers, institutions should offer training on open licensing and consider changes to policies, like recognising open science in career evaluations and allowing extra time to file patents. These steps could make it easier to balance openness with protection.

## Introduction

Open Science (hereafter OS) is a global movement that seeks to make scientific research more transparent, accessible and reproducible. It places emphasis on openly sharing research data, methods and findings to ensure they are accessible to a broad range of stakeholders ( Bornmann *et al.*, 2021; UNESCO, 2021; Vicente-Saez & Martinez-Fuentes, 2018). The core belief of OS is that openness and transparency in the research process can lead to improvements in both the quality and fairness of scientific work ( Chataway *et al.*, 2017) and develop valuable knowledge bases that enable others to build upon them. Concerns such as the reproducibility crisis, particularly in fields like psychology and medicine, have driven increased demands for greater transparency in scientific research ( Anvari & Lakens, 2018; Bausell, 2021; Open Science Collaboration, 2015; Stupple *et al.*, 2019). Furthermore, sharing of data, methods and findings strengthens scientific progress, reduces redundant studies, supports informed policymaking and increases the amount of available scientific resources, a noteworthy aspect in the age of AI.

In recent years, OS, which began as a movement, has developed into a policy agenda, particularly following the UNESCO Recommendation on Open Science in 2021. For instance, in Europe, it has become a core pillar of the Horizon Europe research framework ( Nelhans & Nolins, 2022), and several countries are now integrating OS into their national research policies ( Trinca & Albagli, 2023). This means many funding bodies now mandate and expect that the science they support will be shared and made accessible openly to improve transparency. At the same time, research policies encourage defining and if applicable pursuing intellectual property (hereafter IP) rights to help translate research into practical applications that bring social and economic value, a process often referred to as knowledge valorisation.

IP protects the results of human creativity including inventions, designs, symbols and literary and artistic works when these fulfill certain legal requirements. IP allows research performing organisations (RPOs) to engage with industry to commercialise research, while also generating revenue that can be reinvested into further R&D ( Chen *et al.*, 2024; Hsu & Kuhn, 2023; Subramanian *et al.*, 2022). However, since IP rights are exclusionary by nature, preventing others from using an invention without permission, inherently they appear to be at odds with the principle of sharing promoted by the OS movement. IP and OS, however, stand side-by-side as complementary cornerstones of collaboration and a concerted IP-OS approach lets teams protect what needs to be protected while keeping research outputs FAIR and “ *as open as possible, as closed as necessary.*”

Collaboration is most effective when partners explicitly define both the intended mode of sharing–ranging from internal-only, to a select circle, to full public release–and the timing associated with each stage, creating clarity that builds trust, accelerates the research process, and ultimately lets scientific innovation flourish. While strategic IP-related choices might lead to certain delays in the publication of research results and data, this is compensated by the disclosure and transparency requirements embedded in IP law in the patent system. Apart from strategic delays, IP should not be viewed as a hindrance to OS. In general, an antagonistic relation between IP and OS is due to misconceptions the way the IP system works more than how the system is de jure structured.

In the context of research, tensions between IP and OS arise mainly in two areas. First, copyright, with its “ *all rights reserved*” basis, can restrict access to publications and data thereby limiting its reuse ( Bernier *et al.*, 2023; DuPre *et al.*, 2020; Margoni *et al.*, 2016). As a result, many researchers are unaware of open licenses or how to retain rights. Second, the delayed disclosure that patents often require can lead many researchers to face a situation where they must choose between early sharing and patent protection ( Gans *et al.*, 2017; Walsh & Huang, 2014). This underlying tension between IP and OS has become a significant research policy challenge, as both are seen as important tools for knowledge valorisation.

The conflict is particularly evident in the traditional model of academia–industry collaboration, which relies on IP rights to protect and commercialise research outputs ( Baccigotti, 2024). However, policy debates, particularly within the EU, have increasingly argued that effective IP management is compatible with the principles of OS when supported by clear licensing terms, proper attribution, and time-limited exclusivity ( de la Cueva & Méndez, 2022). In fact, IP law can provide a legal framework that supports the goals of OS when correctly applied ( ALLEA, 2022). The UNESCO (2021) Recommendation also emphasises the importance of a balanced approach, recognising that while scientific knowledge should be as open as possible, access may sometimes need to be limited.

Given these practical challenges, it becomes important to understand how key stakeholders, such as researchers, research managers, knowledge transfer professionals, policymakers and industry partners, perceive the relationship between OS and IP. While policy frameworks increasingly advocate for their compatibility, it is not clear how this synergy plays out in practice or how it is interpreted by those expected to implement it. A better understanding of stakeholder perceptions is essential for informing policy design, improving institutional practices and identifying areas where further support or clarification may be needed. To explore these questions, we conducted a survey to assess stakeholder perceptions on OS and IP, the challenges they encounter and the strategies they have employed (or seen others employ) to balance openness with exclusivity.

While several surveys have been conducted on the adoption, awareness and barriers related to OS practices across disciplines and regions ( Beaudry *et al.*, 2024; El Amin *et al.*, 2023; Hodonu-Wusu *et al.*, 2020; Ng *et al.*, 2024; Ollé *et al.*, 2023; Paret *et al.*, 2022; Rajčáni *et al.*, 2023; Zečević *et al.*, 2021), and others have focused on IP practices within RPOs ( Andersen & Rossi, 2011; Kumar *et al.*, 2021; Payumo *et al.*, 2012; Suominen & Deschryvere, 2024; Wu *et al.*, 2015), to the best of current knowledge, no study has examined stakeholder perceptions at the intersection of IP and OS. Hence, evidence on IP-OS synergy remains limited. This is a critical gap given the growing emphasis on aligning these domains in science policy and practice. Our survey addresses this by exploring challenges and best practices in balancing OS and IP, with the aim of informing more coherent and effective policy and institutional frameworks for knowledge valorisation.

The remainder of the paper is structured as follows. The next section outlines the methods detailing our survey design, sampling frame, questionnaire development and analytical approach. The subsequent results section reports the findings. We then discuss the implications for policy and practice, before concluding with limitations and future research avenues.

## Methods

The study is a part of the Horizon Europe-funded *Unpacking the possibilities of Intellectual Properties for Open Science* (IP4OS) project. We conducted a cross-sectional, online, self-administered survey that was open from 26 March 2025 to 25 May 2025. Participants were recruited using purposive and convenience sampling. Email invitations targeted individuals involved in research, knowledge transfer, policy, publishing and industry. The survey was also shared through mailing lists and online networks including social media platforms (both the project and the authors) such as LinkedIn, Slack, and X/Twitter. Outreach included groups such as the Global Reproducibility Networks, DeSci Foundation, Transfer Alliance Germany, IFLA Volunteers Network in EU, ASTP, de-RSE and the Swedish National Data Service. The survey was hosted on REDCap (Research Electronic Data Capture), a secure and GDPR-compliant data collection platform ( Harris *et al.*, 2009). Informed consent was obtained electronically through the REDCap platform. Participants were informed that, by proceeding with the survey, they confirmed their understanding of its purpose and consented to the use of their responses for research and policy development. Participation was voluntary, and respondents could exit the survey at any time. We did not use any identifying information in the analysis. We received a total of 177 responses. Four of these were partially completed and have been retained. One response contained no completed items and was excluded.

The online questionnaire consisted of four thematic sections that combined closed and open-ended items. It was developed based on an analysis of past systematic reviews on OS, as well as recent reports and policy discussions concerning the relationship between OS and IP ( ALLEA, 2022; Crüwell *et al.*, 2019; de la Cueva & Méndez, 2022; Spellman *et al.*, 2018; Vicente-Saez & Martinez-Fuentes, 2018). The first section, *Background and Professional Context*, collected data on respondents’ professional roles, sector of employment, institutional affiliation and country, along with their familiarity with OS and IP and any direct experience in related policies or agreements. The second section, Perceptions of OS and IP, asked participants to rate their agreement with statements on a 5-point Likert scale, covering whether OS and IP are seen as contradictory or complementary, and whether integration between them is feasible or already occurring in their field. The section also had questions on barriers and enabling factors, focused on perceived challenges such as legal uncertainty, institutional resistance and licensing complexity, as well as factors that may support the effective application of OS and IP practices.

The next section, *Real-World Challenges, Solutions and Best Practices*, collected qualitative accounts of situations where OS and IP were in tension, including how such conflicts were managed, what mechanisms were used and examples of any effective integration models. This section also included questions on policy priorities, where respondents were asked to rate the importance of potential policy interventions, such as mandating open access, providing clear licensing frameworks and offering training for researchers. The final section, *Future Engagement*, asked whether participants were open to follow-up interviews or wished to receive the final report, with optional contact details collected separately to ensure anonymity. An internal pilot test of the questionnaire was conducted within the project team to assess the clarity of terminology, questions and instructions, and to estimate the time required to complete the survey.

Data were analysed using a combination of descriptive statistics and frequency distributions to summarise participant characteristics, perceptions and response patterns across different sections of the questionnaire. Quantitative data were processed using IBM SPSS Statistics (version 29), where counts and percentages were generated where relevant, particularly to explore trends across stakeholder groups and sectors. Figures were prepared externally to improve the clarity of data presentation and to facilitate the visual interpretation of findings. Open-ended responses were reviewed manually to identify illustrative examples; however, no formal qualitative coding or thematic analysis was conducted. Instead, selected quotations and summary observations were used to contextualise the quantitative results. The reporting has been prepared in line with the Checklist for Reporting Results of Internet E-Surveys (CHERRIES) to ensure transparency and rigour ( Eysenbach, 2004). The full protocol is available on Zenodo to support transparency and reproducibility ( Sharma *et al.*, 2025).

## Results

### Respondent profile

**Figure 1.**
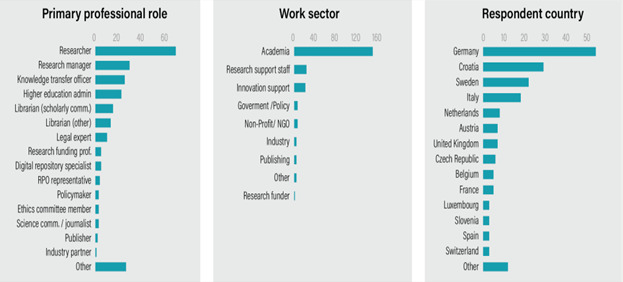
Respondent profile.

Responses were received from 177 individuals. However, as noted in Methods, one submission contained no completed responses and was excluded. The final sample for analysis therefore includes 176 respondents. Of these, four were partial responses; they have been retained in the analysis, and questions with missing data have been noted accordingly. Figure 1 presents the profile of the respondents. Regarding professional roles (n=175; one missing), this was a multiple-response question, as many participants work across more than one role. The largest share of responses is from researchers (68, 38.9%), followed by research managers (29, 16.6%). Knowledge and technology transfer professionals accounted for 25 responses (14.3%), administrators from higher education institutions 22 (12.6%), and librarians (including those involved in scholarly communication and other roles) 28 (16%). Legal experts were identified in 13 responses (7.4%). In terms of sector (n=175; one missing), a substantial proportion of respondents were from the academic sector (149, 85.1%). With respect to country, approximately 57% of responses came from three countries: Germany (31%), Croatia (16%), and Sweden (10%).

### Expertise on IP-OS

**Figure 2.**
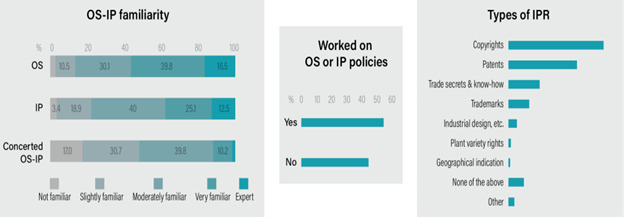
OS IP self-rated expertise and policy experience.

Figure 2 shows respondents’ self-assessed familiarity with IP, OS, and their integrated use (concerted IP-OS approach). A significant majority of respondents (56.3%) reported a high degree of familiarity with OS, rating themselves as “expert” or “very familiar.” A further 30.1% identified as “moderately familiar,” leaving only 13.6% with little to no familiarity. Familiarity with IP was also considerable, though less pronounced than with OS. Over one-third of participants (37.3%) rated their expertise as “expert” or “very familiar.” In contrast, expertise in the concerted use of IP and OS was significantly lower. Only 11.9% of respondents considered themselves experts or very familiar in this area, while a majority (48.3%) reported being “not familiar” or “slightly familiar.” Shifting from familiarity to practical application, most respondents (55%) confirmed having professional experience working with IP or OS-related policies. To identify the specific domains, participants were asked which IP rights they engage with most frequently (n=175, multiple selections permitted). Copyrights were the most cited right (62.9%), followed by patents (45.1%). Other commonly engaged-with rights included trade secrets and know-how (20.6%) and trademarks (13.7%).

### Perception on IP-OS synergy and obstacles

**Figure 3.**
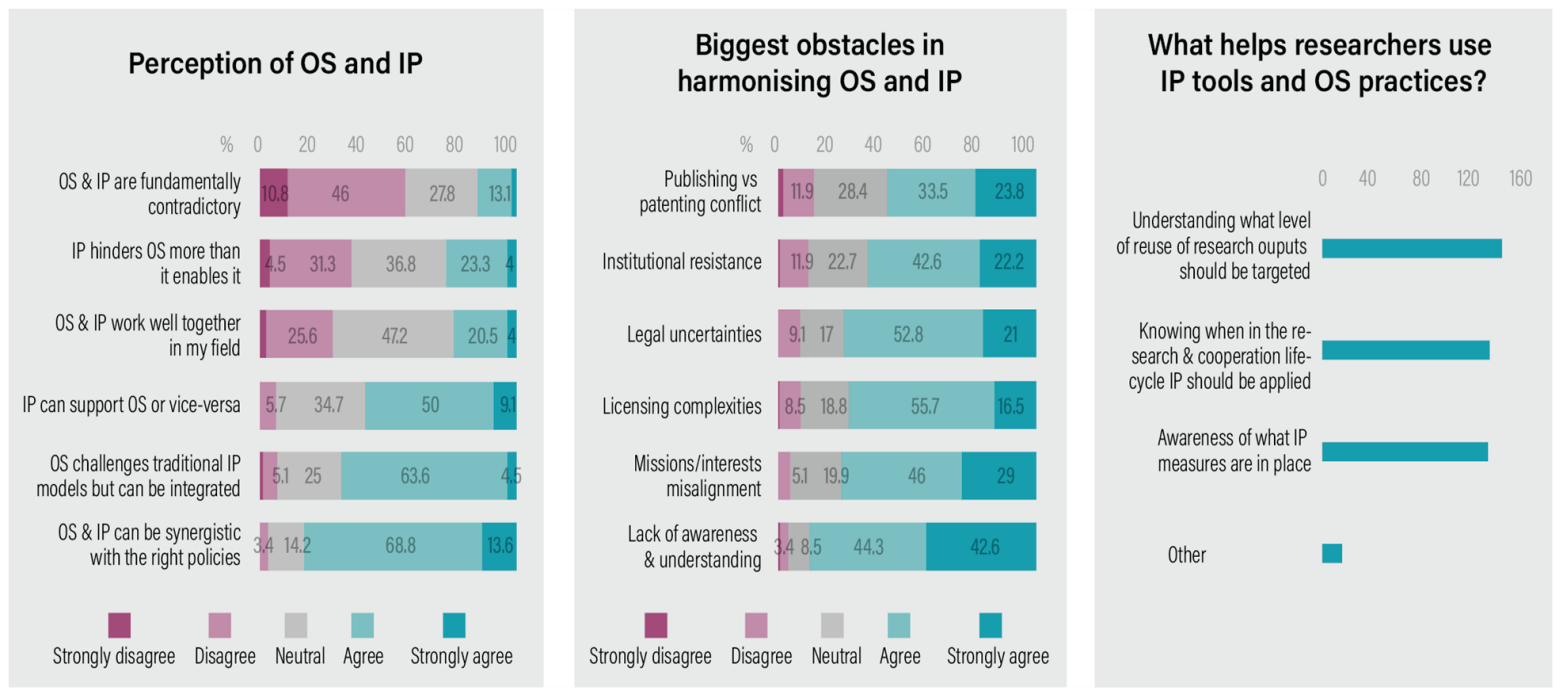
Perception of OS-IP synergy and barriers.

Responses regarding perceptions of IP-OS and their potential synergy reveal a pattern of cautious optimism ( Figure 3). A large majority (82%, agree or strongly agree) of respondents believe that IP and OS can be synergistic with the implementation of appropriate policies, while only 3% strongly disagree with this view. Similarly, there is strong agreement (68%) that OS challenges traditional IP models but can be integrated. Nearly 60% also perceive that IP can support OS, or vice versa. Most respondents (57% strongly disagree/disagree) reject the statement that IP and OS are “fundamentally contradictory”. However, this sentiment is not fully reflected in practice: fewer than 25% feel that IP and OS function well together in their field, with a similar proportion disagreeing with the statement. Opinions on whether “IP hinders OS more than it enables it” remain divided, with approximately 27% agreeing and 37% disagreeing.

Respondents also shared their views on what they consider to be the major obstacles to synergising IP and OS (a multiple-choice question). Knowledge gaps were identified as one of the most significant barriers, with 76% strongly agreeing or agreeing that a lack of awareness and understanding hinders progress. Misalignment between the goals, interests, or missions of collaborators was also rated highly (75% strongly agree/agree), along with legal uncertainties (74%), licensing complexities (72%), and institutional resistance to IP-OS synergy (65%). Notably, although the debate around patenting versus publishing remains relevant, it ranked lowest among the listed obstacles (57%).

In addition to the closed-ended responses, respondents provided open-ended comments identifying further challenges encountered in their respective fields. These reflect a range of legal, organisational, and cultural frictions. For example, some cited a “ *lack of competent persons dealing with IP*” and a “ *lack of learning resources and training*”. Others highlighted structural imbalances, such as the perception that “ *academia on the OS side are amateurs as compared to industry on IP side*” and concerns over the “ *commercial interests of publishers*”. “ *Varying national legal frameworks in the context of international collaborations*” were also mentioned, particularly where “ *RPO interests not always being taken into account sufficiently in legislative efforts*”. Moreover, some pointed to government policies that “ *favour patenting but not access to knowledge*”.

Respondents were asked to rate their opinions on what can support them in selecting appropriate IP tools and OS practices (multiple choice). The results showed that most respondents (n=175) agreed on the importance of understanding the intended level of reuse of research outputs (77%). Equally, 72% of respondents highlighted the importance of knowing at which stage of the research cycle IP should be applied, as well as being aware of existing IP measures. A small number of respondents provided open-ended responses, where they mentioned factors such as “ *awareness of institutional OS policies*” and “ *awareness of the complexities of IP-rights protection and the likelihood of ROI*”. From a practical perspective, it was noted that “ *working together with the KTO of the university*” could be beneficial, since OS and IP often serve different communities and pursue distinct objectives. In addition, proper training and access to suitable tools were also identified as important enablers.

### IP-OS conflict and best practices

**Figure 4.**
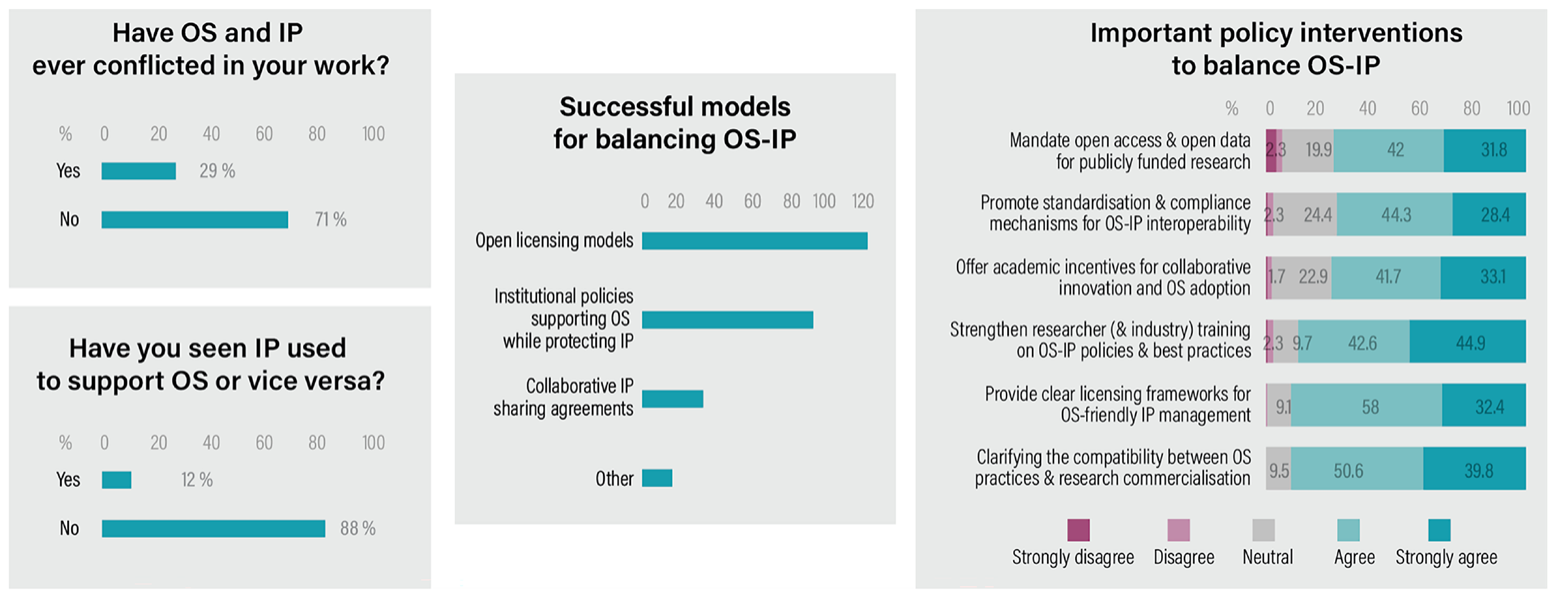
OS-IP conflict experience and policy interventions.

Respondents were asked whether, in their work, IP and OS had ever been in conflict, and whether they had observed any successful models that balanced IP and OS ( Figure 4). A total of 29% of respondents indicated that they had experienced situations in which IP and OS were in conflict. Those who answered *yes* were invited to share the nature of these conflicts, to better understand the practical scenarios where IP and OS hinder synergy. Several tensions are observed from the open-ended responses.

The first issue relates to incompatible timescales. For example, a respondent explains “ *We are forced to publish quickly. On the other hand, for inventions, we must submit a patent first. Writing a patent takes time. Our employer … take 3-4 months to decide, if he wants to apply for a patent or not*.” Such situations are often only managed through embargoes or “ *fast tracked*” filings, which allow researchers to present their work once the legal paperwork has been lodged. Second, respondents highlighted the imbalance of contractual power, which tends to favour publishers and industry. One respondent noted that an “ *industry partner wanted to patent the result*”, which lawfully blocked disclosure until a filing had been secured. Another explained that a firm “ *refused to accept an obligation to share outputs*”, and that these negotiations remain unresolved. Similarly, the transfer of copyrights to journals was seen as a barrier to sharing, as one respondent explained: once “ *copyright is transferred to publishers, …. unable to provide open access*”. A third issue concerns limited IP literacy within academia, which can result in self-inflicted conflicts. Respondents described instances where publications about relevant results were submitted “ *without weighing the possibility of protecting them*”, and without prior consultation with Knowledge Transfer Offices (KTOs).

Shifting from conflict to synergy, respondents were asked whether they had observed, in their context, a situation where IP was used to support OS or vice versa. Only 12% reported having witnessed such instances. To explore the nature of these practices, those respondents were asked to elaborate through open-ended responses, which resulted in several informative examples.

One respondent described a case where a public–private group provided an “ *open probe for the WDR5 protein*” so that other laboratories could validate it. Once flaws were identified, the probe was revisited and a “ *modified molecule*” was “ *patented… and licensed at the preclinical stage*”, illustrating how openness was initially used to improve the output, followed by protection through IP. Similar examples were provided from the software sector. For instance, respondents mentioned how software consortia adopt permissive licences. One noted that the LLVM compiler project uses “ *the Apache licence… allowing corporations to take the code and use it privately without any conflict*”, while firms remain aligned with the upstream code to avoid costly divergence.

Strategically timing the publication and patent application also emerged as a successful strategy for aligning OS and IP. Several partnerships follow a “ *patent first, publish later*” approach as one respondent stated, “ *first I patented. Once I presented the provisional presentation, the manuscript was submitted*”, while another described a collaboration where filing was fast-tracked so an academic could present at a conference. Another respondent outlined a case where different research outputs were used strategically: “ *Publishing data and software in open source with no commercial license while using the software in a patent to support the resolution of a technical issue in a manufacturing machine*.” This shows how OS and IP can be applied in parallel to different components of the same research. Legislative mechanisms were also cited as enablers in situations where rights holders were unresponsive. For example, a respondent from Sweden reported, “ *We have used paragraph 23 of Swedish Copyright Law, to reuse copyrighted material, when commercial rights holders have not answered permission requests.*”. The applicability of such legal provisions have to be independently verified.

An open-ended question was also included to explore whether respondents had observed any successful alignment of OS and IP in specific sectors beyond their area of work. One respondent highlighted the principle “ *as open as possible, as closed as necessary*” as an effective approach. However, they noted that its success depends on institutional support, stating that this is only feasible when organisations “ *support the researchers … by adequate employees*” such as IP managers and legal staff. Another respondent pointed to the “ *open governmental data initiative in Switzerland*”, which mandates that all publicly funded data must be open source. This was presented as a structural approach that builds alignment between OS and IP. Practical tools and frameworks were cited as helpful in achieving synergy. One respondent referred to “ *the EOSC checklist for researchers … an excellent framework and starting point that encourages researchers to consider both IP and data protection throughout the life of a project*.” Other initiatives mentioned included open-source models that have led to commercial success. For example, “ *the Opentrons ecosystem is a nice example of open source-based projects that have led to a viable private business, although not all open science values have been preserved*.” Another example was the Structural Genomics Consortium, which “ *does not seek patents but its private sector partners use that knowledge to develop proprietary products*.”

Respondents were also asked to select, from a predefined list, what they considered to be successful models for aligning OS and IP (multiple choice; n=175). Nearly 75% of those who answered this multiple-choice question selected ‘open licencing models (eg., Creative Commons)’ as a successful pathway, indicating that the use of available Creative Commons (CC) licences is widely seen as an effective method for balancing proprietorship and openness. Similarly, ‘institutional policies supporting OS while protecting IP’ was selected by 56% of respondents. In contrast, only 20% selected ‘collaborative IP sharing agreements’, such as patent pools and FRAND models, as successful approaches. In addition, 16 respondents provided open-ended responses identifying other models they considered effective.

One respondent, for example, noted that at social sciences data archives, they “ *use special licences that restrict access to defined purposes, but still allow data reuse*”, highlighting how purpose-limited licences can balance access and control. Another example cited the Horizon Europe consortiums, where “ *partners have an obligation to use open science practices, but must always consider IP first*”, ensuring that both OS and IP considerations are embedded from the outset of the project. Several respondents, however, expressed the view that a universally successful model does not yet exist. Comments included, “ *have yet to see a successful model*” and “ *Nothing widely successful at global scale*”.

Process innovations were mentioned as possible enablers of IP-OS synergy. These included “ *blockchain-based consortia models (e.g. EU funded PharmaLedger)*”, “ *Open science partnership agreements”, “Patent First, Publish Later*”, and the use of “ *embargo periods in institutional open access and/or research data repositories*”. Finally, legal mechanisms beyond traditional patents were also raised. Respondents referred to “ *secondary publication rights legislations*” and “ *Preferential (free) licensing models for publicly owned entities and non-for profit use ...*” as possible instruments to support alignment between OS and IP.

Last, we asked the respondents to rate their opinion on the various policy interventions and whether they believed these would be effective in balancing OS and IP. Respondents showed the strongest support for three specific interventions: (1) clarifying the compatibility between OS practices and research commercialisation (91% agree or strongly agree), (2) providing clear, licensing frameworks for OS friendly IP management (90%), and (3) strengthen researcher and industry training on OS IP policies and best practices (88%). Together, these three policy interventions received highest support.

## Discussion

Our survey finds that an overwhelming proportion of respondents are aware of OS and its practices. This is in line with other studies that have found the same, where researchers are aware of OS largely due to their use of open access practices ( Grattarola *et al.*, 2024; Ollé *et al.*, 2023). High levels of familiarity with IP were also expected, as many RPOs now invest in setting up systems for managing academic inventions ( Halilem *et al.*, 2017). However, both OS and IP have long existed as two parallel tracks with little interaction and are often considered to be at odds. This is evident in the large proportion of respondents in our survey who were less familiar with a concerted OS-IP approach, despite more than half having worked on OS or IP policies in their work.

The results on perceptions of OS and IP as being complementary or conflicting show that respondents largely believe coexistence between the two is very much possible, but current practical arrangements are still unable to deliver on how to achieve this synergy. Therefore, one of the significant tasks is to devise policies and guidelines that help stakeholders realise this synergy, as philosophical opposition to coexistence is not the issue. Policy reports have asserted that the idea of OS and IP being incompatible is false, and that OS components need to be examined through an IP lens to ensure proper application ( de la Cueva & Méndez, 2022). A recent study with neuroscientists in Canada also concluded that more patent education, support for data sharing, and investment in digital infrastructure could be one way to achieve a hybrid OS-IP model ( Nuechterlein *et al.*, 2023).

Fewer than a third of respondents from our survey have experienced a conflict between OS and IP in their work. The open-ended responses from these individuals, aimed at understanding the nature of this conflict, reveal various issues that are manageable with the right policies. For instance, the debate of publishing versus patenting has been a long-standing issue in academia and is particularly problematic for early career researchers, who are expected to publish. Hence, timely sequencing of patent applications in such cases, along with prompt communication with the relevant KTOs, could offer appropriate solutions. Some policy advocacy has also focused on introducing a well-defined grace period of at least one year to allow publication before filing a patent ( ALLEA, 2022) while others have also discussed its pros and cons and have noted that any reform would require a careful balancing of the interests of the innovators and other stakeholders ( EPO, 2015).

Similar concerns have been raised in industry–academia collaborations, where industry partners tend to favour secrecy and withholding of results, while academic partners are more inclined towards sharing. Several research fields, especially in early-stage research, have begun to experiment with Open Science Partnership (OSP) models ( Gold *et al.*, 2019). These models require, from the outset, open sharing of all research outputs and not allowing IP claims. However, there is uncertainty about whether research generated through OSPs will ultimately lead to commercialisation, and whether firms will be willing to engage without IP control ( Norn & Ramos-Vielba, 2025).

Regarding publication outputs, one of the major conflicts in the OS-IP synergy concerns the transfer of rights to publishers, which results in researchers losing access to their own work. Several EU countries have incorporated secondary publication rights into their copyright laws, particularly for publicly funded research. Current proposals within EU copyright reform now aim to extend this into an EU-wide secondary publication right to support open access to scientific outputs ( European Commission, 2024).

The survey results also reveal that only a small number of respondents reported having witnessed a successful case of OS-IP synergy. The anecdotal responses to our open-ended question highlighted the publishing versus patenting dilemma, the sharing of data by researchers, and the publication of data and software in open source. While several such examples do exist in practice, where OS and IP have been strategically combined, their sector-specific nature means these successes have not transferred easily to other fields, resulting in limited awareness or acceptance.

For instance, the Montreal Neurological Institute’s OS initiative commits to open data, open materials, and no patents ( Gold, 2016), with the aim of disseminating university knowledge and reducing barriers to upstream research. While this model may have worked well in the context of Canada, in many other sectors, industries and regions, a commitment to avoiding IP claims could make collaborations less attractive to firms. Furthermore, some of these practices operate in different legislative frameworks and require discussion on their transferability to different contexts. Therefore, another recommended approach is to decide from the outset how IP and OS will be used, in a way that is acceptable to all partners and avoids creating barriers later in the research process. In terms of publications, the use of preprints to share early results is becoming more popular beyond those disciplines that have historically adopted preprints to share their findings ( Alfonso & Crea, 2023).

Open licensing models have been rated by respondents as one of the most successful models for balancing OS and IP. In this context, CC licences have gained rapid acceptance within the creator community, as they make copyrighted works more accessible for use and distribution ( Katz, 2005). Similar licences exist for software, such as the MIT licence, which allows free use, including for commercial purposes, without requiring signatures or licence fees ( Saltzer, 2020). However, open-source licences are not without restrictions; each licence sets out what users may do, and authors always retain ownership ( Hahn, 2014). Therefore, the availability of different licences and the importance of choosing the right one based on the intended purpose of the IP and the goals of knowledge sharing become crucial.

### Practical and policy implications

Our study has both practical and policy implications. In practice, it is important that researchers are encouraged and supported in filing patent applications in a timely manner, allowing them to share findings and other outputs openly without compromising patenting possibilities. In academia-industry collaborations, it is essential that agreements or guidelines on how OS and IP will be handled are established from the start, covering all aspects of the research partnership. Most importantly, institutions should invest in practical training for researchers at all career stages on how to align OS practices with IP considerations, as selecting the appropriate licensing tools can be complex.

From a policy perspective, OS practices should be actively incentivised by integrating them into career advancement frameworks, including criteria for promotion and awards. At present, research assessments tend to prioritise metrics such as patents and publications, but institutionally, OS should also be recognised as a component of career progression. Moreover, it is important to engage with stakeholders on the potential introduction of grace periods for patent filing and balancing the interests of all stakeholders ( EPO, 2015). Such provisions, as implemented in some jurisdictions, would enable researchers to publish openly while retaining the right to file a patent within a defined time window, supporting both openness and exclusivity.

### Limitations

Our study has limitations that are important to acknowledge. First, the sample size is relatively small, which limits the generalisability of the findings. The aim of the survey was to provide insights into perceptions of IP-OS synergy, and the results should not be considered representative of all stakeholders and should therefore be interpreted with caution. Second, our use of an online questionnaire might have resulted in response bias and recall bias. In such surveys, respondents may answer in ways which they think are desirable or may not have accurately recalled past experiences, particularly on questions requiring reflection on conflict or best practices in their field on OS IP synergy. Last, our sampling approach was purposive and based on convenience, relying on outreach to known networks. This may have led to an overrepresentation of individuals already engaged with or aware of issues related to IP-OS, potentially impacting diversity of perspectives, and hence follow-up studies with broader and more representative samples are needed to validate and expand on our findings.

## Conclusion

Our study showed that the synergistic use of OS and IP for knowledge valorisation remains uncommon, despite stakeholders being optimistic that complementarity between the two can be achieved. The absence of appropriate policy tools for this purpose makes the community somewhat cautious about how best to realise it. Lack of awareness and understanding of how such synergy can be achieved is one of the major obstacles to harmonising OS and IP, alongside challenges such as differing goals among partners and collaborators in multi-partner projects. The role of KTOs and OS ambassadors is particularly important in these contexts, and it is equally vital for researchers to maintain sustained engagement with them. Such interaction can support RPOs in better understanding the compatibility between OS and research commercialisation, as well as understanding the complexity of choosing the appropriate licensing tools. It should also be noted that there are differences in national legal frameworks across EU member states and this may pose certain challenges to the development of a fully synergistic approach to IP and OS.

## Ethics and consent

The proposed study has been performed in accordance with the principles and requirements stated in the Declaration of Helsinki. Ethical standards were upheld by ensuring voluntary participation, informed consent, data anonymity, and secure data handling. Participants were clearly informed about the study’s purpose, confidentiality measures and their right to withdraw at any time. No sensitive personal data were collected, and optional identifiers were stored separately to prevent reidentification. Data were analysed and reported in aggregate to protect individual privacy. The research team adhered to GDPR and other relevant data protection laws.

As the research posed minimal risk, involved no sensitive personal data, and participation was entirely voluntary with informed consent, ethics committee review was not required nor possible under the applicable Swedish legislation. The only personal information collected was the respondent’s country, institutional affiliation, and email address, which were included solely for potential future contact and were not used in the analysis.

## Data Availability

Zenodo: *IP4OS Best Practices Survey Dataset.* https://doi.org/10.5281/zenodo.15728364 ( Sharma & Nilsonne, 2025a). The project contains the following underlying data: •  IP4OS Best Practices Survey Dataset. (This dataset contains anonymised survey responses collected via REDCap for a study on best practices in IP and OS research. The dataset includes fully de-identified responses and a data dictionary describing all variables and coding used in the survey.) Data are available under the terms of the Creative Commons Zero v1.0 Universal license. Zenodo: *IP4OS Survey Questionnaire: Best practices for Open Science (OS) & Intellectual Property (IP).* https://doi.org/10.5281/zenodo.15916145 ( Sharma & Nilsonne, 2025b). The project contains the following extended data: •  IP4OS Survey Questionnaire: Best practices for Open Science (OS) & Intellectual Property (IP). (This file contains the blank version of the questionnaire used to collect the responses) Data are available under the terms of the Creative Commons Attribution 4.0 International license.
