# Supplementary material for: Stakeholders’ perceptions of the synergy between intellectual property and open science: A cross-sectional survey
Source: Open Res Eur. 2025 Aug 7;5:229. [Version 1] doi: 10.12688/openreseurope.20782.1 (PMC13234549; doi:10.12688/openreseurope.20782.1)
Supplement: Peer review history

# Reviewer report: Reviewer response for version 1

**DOI:** 10.21956/openreseurope.22486.r74360

**Published:** 2026-06-03

**Authors:** Maria Theresa Norn

**Affiliations:** 1 Aarhus University Department of Political Science (Ringgold ID: 193584), Aarhus, Central Denmark Region, Denmark

This paper reports findings from a cross-sectional online survey of 176 respondents (primarily academic researchers and research managers in Europe) examining how stakeholders perceive the relationship between intellectual property (IP) and open science (OS). A majority of respondents describe IP and OS as potentially complementary and even mutually reinforcing, yet few have witnessed this playing out in practice. Respondents point to barriers such as knowledge gaps, legal uncertainty, licensing complexity, and misaligned goals among collaborators. The authors conclude that greater guidance, training, and policy support are needed.

**Major comments**

 1. Conceptual clarity and framing. The paper focuses on the relationship between OS and IP using terms such as "synergy", "complementarity", "integration", and "harmonisation". However, these concepts are never clearly defined and are often used interchangeably, despite referring to somewhat different phenomena. In addition, the paper does not sufficiently explain why OS and IP might be considered complementary in the first place, or through which mechanisms such complementarity would emerge. I would encourage the authors to develop a clearer conceptual framework at the outset of the paper, including more explicit definitions and a discussion of different possible relationships between OS and IP. On a related note, the manuscript tends to assume the desirability and feasibility of OS-IP complementarity from the outset. A somewhat more neutral framing of the topic would strengthen the paper and allow the empirical findings to speak more directly to the question of whether, and under what conditions, complementarity is possible.

 2. Does the study examine perceptions or practice? A key strength of the paper is that it provides insight into stakeholder perceptions of the relationship between OS and IP. However, at several points in the discussion and conclusion, the manuscript moves beyond perceptions and towards claims about practices, successful approaches, and policy solutions. I would encourage the authors to more clearly distinguish between *perceptions *and *evidence *of what works. The survey provides valuable insight into stakeholder beliefs and experiences but cannot establish which approaches are in fact effective in enabling OS-IP integration.

 3. Engagement with the literature. I was surprised by the relatively limited engagement of the paper with literature streams on knowledge valorisation, technology transfer, open innovation, and the well-researched question of how openness and appropriability are balanced. Expanding the review would not only strengthen the grounding of the paper but would also help clarify why the question of OS-IP complementarity is important and how this study contributes to existing knowledge.

 4. Sampling. The authors acknowledge limitations relating to sample size and sampling strategy. However, I believe the implications of these limitations deserve deeper discussion. The survey was distributed through networks that are already strongly engaged with open science and related topics. This may have resulted in a sample that is both more knowledgeable about OS and more positively disposed towards it than the broader research population. Furthermore, the sample is overwhelmingly academic and geographically concentrated (esp. Germany, Croatia, and Sweden). Given this, I would encourage the authors to reflect more explicitly on how the composition of the sample may have shaped the findings and the extent to which the term "stakeholders" accurately reflects the respondent population.

 5. Survey framing and interpretation. Several survey items appear to be framed around the assumption that OS-IP synergy is both desirable and achievable. For example, respondents are asked about barriers to harmonising OS and IP, policy interventions to support alignment, and successful models of integration. This framing may have encouraged respondents to view complementarity as the primary objective rather than considering whether tensions or trade-offs between openness and protection are sometimes unavoidable. I would consider acknowledging this possibility as a limitation. Also, it was not clear to me that respondents shared a common understanding of what "OS-IP synergy" is, which could be an additional source of variation in responses that warrants discussion.

 6. Policy implications. The recommendations presented are sensible and aligned with survey responses. But I would consider adopting a somewhat more cautious tone when presenting these recommendations. While the survey identifies perceived barriers and stakeholder preferences, it does not provide evidence regarding the effectiveness of specific interventions such as training programmes, grace periods, licensing frameworks, or changes to academic evaluation systems. Distinguishing more clearly between stakeholder support for these measures and evidence of their effectiveness would strengthen the discussion.

 Minor comments

 7. I would have liked to see more detailed exploration of the data, but given the small no. of observations, things like differences between respondent profiles, country-level variation, and the impact of familiarity with IP-OS on perceptions would be difficult to pursue. Still, any deeper insights into the data that are possible and meaningful to generate would be interesting.

 8. The authors state that open-ended responses were not subject to formal coding or thematic analysis, which surprised me given the focus of the paper. Was this for lack of time or because the open-ended responses did not contain valuable information?

 9. From the opening of the paper: The discussion of "as open as possible, as closed as necessary" would benefit from further elaboration. While this principle is well established within OS and European R&I policy (esp. with regards to research security), it is less clear how it specifically informs decisions regarding IP. A short explanation of how the principle applies in this context would strengthen the argument.

 Overall assessment: This paper addresses a timely and important topic at the intersection of OS and IP. The survey provides useful exploratory insights into how different actors perceive the relationship between OS and IP and highlights a number of challenges that merit further investigation. The paper's main contribution lies in documenting these perceptions and opening an important discussion about the conditions under which openness and IP-based appropriability can coexist. At the same time, the manuscript would benefit from revision, including a stronger conceptual framing and a more cautious distinction between stakeholder perceptions and evidence on effective practice.

If applicable, is the statistical analysis and its interpretation appropriate?

Partly

Is the study design appropriate and is the work technically sound?

Partly

Is the work clearly and accurately presented and does it engage with the current literature?

Partly

Are the conclusions drawn adequately supported by the results?

Partly

Are sufficient details of methods and analysis provided to allow replication by others?

Yes

Are all the source data and materials underlying the results available?

Yes

Reviewer Expertise:

Science and innovation policy; university policy; open innovation in science; open science partnerships; university-industry collaboration etc.

I confirm that I have read this submission and believe that I have an appropriate level of expertise to confirm that it is of an acceptable scientific standard, however I have significant reservations, as outlined above.

# Reviewer report: Reviewer response for version 1

**DOI:** 10.21956/openreseurope.22486.r74358

**Published:** 2026-05-26

**Authors:** Taewoo Roh

**Affiliations:** 1 Hanyang University, Seoul, South Korea

1. This manuscript addresses an important and timely topic by examining stakeholders’ perceptions of the relationship between intellectual property and open science. The topic is highly relevant given the increasing policy emphasis on knowledge valorisation, open access, research data sharing, and university-industry collaboration. However, the manuscript remains primarily descriptive and does not yet provide a sufficiently strong theoretical or analytical contribution to justify indexing.

 2. The manuscript begins from the premise that IP and OS can be synergistic, but the core concept of “synergy” is not clearly theorized. At several points, synergy appears to mean compatibility, coexistence, sequential use, legal alignment, institutional coordination, or practical problem-solving. These meanings are not equivalent. The paper needs a clearer conceptual definition of what IP-OS synergy means and how it differs from simple non-conflict or pragmatic compromise.

 3. The introduction provides useful background on OS, IP, patents, copyright, open licensing, and knowledge valorisation, but it does not develop a strong research question. The paper states that it aims to explore stakeholder perceptions, challenges, and strategies, but the resulting study is framed more as an exploratory policy survey than as a theoretically grounded research article.

 4. The literature review is too narrow for the breadth of the research topic. IP and OS are connected to broader debates on public goods, scientific commons, university technology transfer, academic entrepreneurship, knowledge valorisation, open innovation, and responsible research policy. The current manuscript references some relevant policy documents and empirical studies, but it does not sufficiently engage with the larger scholarly debates that would allow the study to make a meaningful academic contribution.

 5. The manuscript claims that “no study has examined stakeholder perceptions at the intersection of IP and OS.” Even if this is accurate, novelty based on an underexplored empirical intersection is not sufficient. The authors need to explain what theoretical insight can be generated from examining this intersection and why stakeholder perception data are the appropriate way to advance that insight.

 6. The survey design is useful for collecting exploratory perceptions, but the sampling strategy raises serious concerns. The study relies on purposive and convenience sampling through professional networks, mailing lists, social media, and project-related channels. As a result, the sample is likely to overrepresent individuals already interested in OS or IP, which limits the interpretability and generalisability of the findings.

 7. The respondent profile confirms this concern. The manuscript reports that 85.1% of respondents are from academia, and approximately 57% of responses come from Germany, Croatia, and Sweden. This strong concentration by sector and country makes it difficult to claim that the findings reflect broader stakeholder perceptions across research-performing organisations, policymakers, industry partners, publishers, funders, and legal actors.

 8. The paper frequently uses the term “stakeholders,” but the actual stakeholder composition is uneven. Researchers and academic actors dominate the sample, whereas industry partners, policymakers, research funders, publishers, and legal experts appear much less represented. Since IP-OS tensions often emerge precisely in cross-sector interactions, this imbalance weakens the paper’s ability to draw conclusions about stakeholder synergy.

 9. The analysis remains largely descriptive. The paper reports percentages, frequency distributions, and selected quotations, but it does not conduct more systematic comparisons across stakeholder groups, sectors, countries, or levels of expertise. Given the survey structure, the paper could have examined whether perceptions differ by role, sector, IP familiarity, OS familiarity, or direct experience with IP-OS conflict.

 10. The qualitative responses are potentially valuable, but the manuscript states that no formal qualitative coding or thematic analysis was conducted. Instead, selected quotations are used illustratively. This limits the analytical value of the open-ended responses, especially because many of the manuscript’s most interesting claims concern barriers, conflicts, and best practices that would benefit from systematic qualitative analysis.

 11. The results identify several important barriers, such as knowledge gaps, legal uncertainty, licensing complexity, institutional resistance, and misaligned collaborator goals. However, these findings are not sufficiently integrated into a conceptual framework. The manuscript would be stronger if it classified these barriers into individual-level, organisational-level, legal-institutional, and inter-organisational dimensions.

 12. The conclusions and policy recommendations sometimes appear stronger than the evidence allows. For example, the manuscript recommends early agreement on IP-OS approaches, targeted training, recognition of OS contributions in career evaluation, and grace periods for patent filing. These recommendations are reasonable, but the descriptive survey evidence does not fully establish which interventions are most effective or under what conditions they would work.

 13. The manuscript should engage more deeply with the public domain as an alternative to the IP-OS binary. Some scientific communities deliberately avoid patenting to keep research outputs openly available, while others rely on defensive publication, open licensing, or commons-based governance. Without addressing the public domain and knowledge commons more explicitly, the paper’s framing of IP and OS remains incomplete.

 14. The manuscript’s practical contribution is clearer than its scholarly contribution. It provides a useful snapshot of how a network of mostly European, academically oriented respondents perceive IP-OS tensions. However, this is not enough for a strong research article unless the authors show how these perceptions refine existing theories of open science, knowledge commons, university technology transfer, or science policy governance.

If applicable, is the statistical analysis and its interpretation appropriate?

Partly

Is the study design appropriate and is the work technically sound?

No

Is the work clearly and accurately presented and does it engage with the current literature?

No

Are the conclusions drawn adequately supported by the results?

Partly

Are sufficient details of methods and analysis provided to allow replication by others?

No

Are all the source data and materials underlying the results available?

Partly

Reviewer Expertise:

My areas of research are in open innovation, research data management, and nonmarket strategy, with a particular interest in how intellectual property and open science can be aligned to support transparent and reproducible research.

I confirm that I have read this submission and believe that I have an appropriate level of expertise to state that I do not consider it to be of an acceptable scientific standard, for reasons outlined above.

# Reviewer report: Reviewer response for version 1

**DOI:** 10.21956/openreseurope.22486.r58603

**Published:** 2025-09-10

**Authors:** Luke McDonagh

**Affiliations:** 1 London School of Economics and Political Science Law School, Houghton St, London, England, UK

This interesting paper investigates how various stakeholders view the relationship between intellectual property rights (IP) and open science practices (OS), observing that although there exists enthusiasm about their possible complementary nature, real-world coordination frequently falls short. The researchers implemented a study involving 177 participants, mostly from European countries, who work in research or research administration to gauge contemporary perspectives. The paper shows that while respondents believe IP and OS can work together, genuine successful combination occurs infrequently, mainly because of insufficient clear organisational direction, inadequate knowledge of open licensing frameworks, and conflicting goals among partners.

 The authors suggest that improvements could come from an upfront approach to methodologies and specialised education for academics. This could promote a more successful equilibrium in scholarly activities.

 The researchers recognise constraints, such as a limited, selective sample that could impact how broadly these findings apply.

 In its conclusion, the publication calls for regulatory measures and assistance to close the divide between theoretical compatibility and real-world execution.

 Although a limited study, overall the paper presents a useful snapshot of this intriguing question of whether OS and IP can be reconciled. 

 A future study should consider in more detail the value of the public domain, rather than simply a binary between IP and OS. Some scientists choose not to patent in order to keep their research findings in the public domain, and there is more to say about the usefulness of this option.

If applicable, is the statistical analysis and its interpretation appropriate?

Yes

Is the study design appropriate and is the work technically sound?

Yes

Is the work clearly and accurately presented and does it engage with the current literature?

Yes

Are the conclusions drawn adequately supported by the results?

Yes

Are sufficient details of methods and analysis provided to allow replication by others?

Yes

Are all the source data and materials underlying the results available?

Yes

Reviewer Expertise:

Intellectual Property Law

I confirm that I have read this submission and believe that I have an appropriate level of expertise to confirm that it is of an acceptable scientific standard.

# Reviewer report: Reviewer response for version 1

**DOI:** 10.21956/openreseurope.22486.r58600

**Published:** 2025-08-26

**Authors:** Raj Kumar Yadav

**Affiliations:** 1 Central University of Punjab, Bathinda, Punjab, India

**Constructive Suggestions for Improvement**

**Clarity and Structure**: Some parts of the paper could be reorganized for better flow. Shorter paragraphs and clearer transitions would enhance readability.

**Literature Engagement**: Expand the literature review to incorporate more recent publications, particularly in the last 3–5 years.

**Data Transparency**: Where possible, provide access to the underlying data and materials in supplementary files or an accessible repository.

**Conclusions**: Revisit the conclusions to ensure they are proportionate to the evidence presented. Avoid generalizations that are not directly supported by the findings.

**Overall Recommendation**

 The article addresses an important subject and has potential, but requires revision to meet the standards of scientific soundness and replicability. I recommend **major revisions** focusing on methodological detail, engagement with recent literature, and alignment of conclusions with presented evidence.

If applicable, is the statistical analysis and its interpretation appropriate?

Not applicable

Is the study design appropriate and is the work technically sound?

Partly

Is the work clearly and accurately presented and does it engage with the current literature?

Partly

Are the conclusions drawn adequately supported by the results?

Partly

Are sufficient details of methods and analysis provided to allow replication by others?

Partly

Are all the source data and materials underlying the results available?

Partly

Reviewer Expertise:

Intellectual Property Rights

I confirm that I have read this submission and believe that I have an appropriate level of expertise to confirm that it is of an acceptable scientific standard, however I have significant reservations, as outlined above.
